# INTEGRATING FRAILTY AND COGNITIVE PHENOTYPES: THEORY, MEASUREMENT, APPLICATIONS

**DOI:** 10.1093/geroni/igz038.1464

**Published:** 2019-11-08

**Authors:** Qianli Xue, Brian Buta, Lina Ma, Meiling Ge, Michelle carlson

**Affiliations:** 1 Department of Medicine, School of Medicine, Johns Hopkins University, Baltimore, Maryland, United States; 2 Department of Medicine Division of Geriatric Medicine and Gerontology, School of Medicine, Johns Hopkins University, Baltimore, Maryland, United States; 3 Department of Geriatrics, Xuanwu Hospital, Capital Medical University, Beijing, Beijing, China (People’s Republic); 4 The Center of Gerontology and Geriatrics (National Clinical Research Center for Geriatrics), West China Hospital, Sichuan University, Chengdu, Sichuan, China (People’s Republic); 5 Department of Mental Health, Bloomberg School of Public Health, Johns Hopkins University, Baltimore, Maryland, United States

## Abstract

The fact that frailty and cognitive impairment are associated and often coexist in older adults has led to the popular view of expanding the definition of frailty to include cognitive impairment. However, there is great variability in approaches to and assumptions regarding the integrated phenotypes of physical frailty and cognitive impairment. By reviewing the theoretical underpinnings of three integrated phenotypes of physical and cognitive impairments, this talk advocates the incorporation of biological theories in phenotype development that helps determine shared and distinct pathways in the progression to physical and cognitive impairments.

